# Recent Advances on the Use of Biochemical Extracts as Filaricidal Agents

**DOI:** 10.1155/2013/986573

**Published:** 2013-11-05

**Authors:** Nazeh M. Al-Abd, Zurainee Mohamed Nor, Abdulelah H. Al-Adhroey, Anwar Suhaimi, S. Sivanandam

**Affiliations:** ^1^Department of Parasitology, Faculty of Medicine, University of Malaya, 50603 Kuala Lumpur, Malaysia; ^2^Department of Rehabilitation Medicine, Faculty of Medicine, University of Malaya, 50603 Kuala Lumpur, Malaysia

## Abstract

Lymphatic filariasis is a parasitic infection that causes a devastating public health and socioeconomic burden with an estimated infection of over 120 million individuals worldwide. The infection is caused by three closely related nematode parasites, namely, *Wuchereria bancrofti*, *Brugia malayi*, and *B. timori*, which are transmitted to human through mosquitoes of *Anopheles*, *Culex*, and *Aedes* genera. The species have many ecological variants and are diversified in terms of their genetic fingerprint. The rapid spread of the disease and the genetic diversification cause the lymphatic filarial parasites to respond differently to diagnostic and therapeutic interventions. This in turn prompts the current challenge encountered in its management. Furthermore, most of the chemical medications used are characterized by adverse side effects. These complications urgently warrant intense prospecting on bio-chemicals that have potent efficacy against either the filarial worms or thier vector. In lieu of this, we presented a review on recent literature that reported the efficacy of filaricidal biochemicals and those employed as vector control agents. In addition, methods used for biochemical extraction, screening procedures, and structure of the bioactive compounds were also presented.

## 1. Introduction

**Figure 1:**
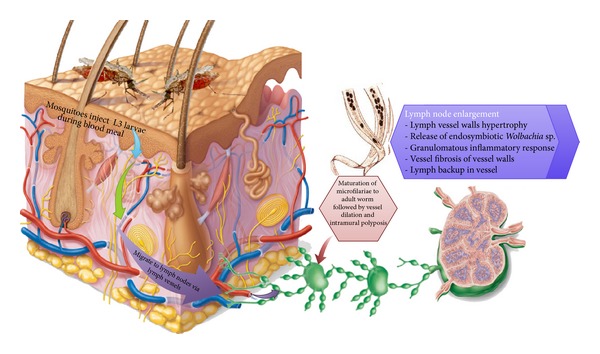
Inflammatory response in lymphatic filariasis.

Lymphatic filariasis is a disease that is caused by parasitic helminthes, namely, *Wuchereria bancrofti*, *Brugia malayi*, and *B. timori*. The parasites are transmitted by several mosquito species [1, 2], and the disease is reported to constitute serious public health and socioeconomic issues. In fact, it is said to be a major cause of acute and chronic morbidity in humans within tropical and subtropical areas of Africa, Asia, the Western Pacific, and some parts of the Americas [3]. It has been characterized with long-term infection through suppression of host immunity [1]. The pathogenesis of lymphatic filariasis is linked to host inflammation invoked by the death of the parasite (Figure 1), resulting in an altered lymphatic system and the abnormal enlargement of body for example, hydrocoele, lymphedema, and elephantiasis, causing pain and severe disability. The filarial species that infect people are known to coexist in a mutualistic endo-symbiotic relationship with *Wolbachia* bacteria, which are reported to be essential for the growth, development, and survival of the nematode hosts [1]. These endosymbionts are said to be among the factors that contribute to the inflammatory effect of this disease [1]. According to World Health Organization (WHO) fact sheets, more than 1.3 billion individuals in 72 countries worldwide are threatened by lymphatic filariasis, with over 120 million individuals being currently infected, and about 40 million being disfigured and incapacitated by the disease [2].

Currently, the chemotherapeutic drugs used to treat filariasis include doxycycline therapy, which targets the endosymbionts, delivers macrofilaricidal activity and improves pathological outcomes. Interestingly, the drug is said to be effective, even when used as monotherapy [1]. Combined therapeutic dosage of diethylcarbamazine (DEC), ivermectin, and albendazole effectively reduces microfilariae in blood [1, 4]. Unfortunately, most of these chemical medications are characterized by adverse side effects. For example, DEC has been around and in use since 1947 and is considered a good medication against microfilarial infection. Regrettably, this drug is reported to have a detrimental side effect [4]. Ivermectin, though reported to significantly lower the concentration of the microfilaria in the blood, was found to be associated with recurrence of microfilaraemia after treatment [4]. Hence, considering the public health and socioeconomic burden due to this disease, research on exploration and development of alternative therapeutic drugs, especially placid and/or less hazardous drugs of natural “organic” origin, is highly recommended.

Among the complementary alternative therapies that have been investigated is the exploration of biomedicine (botanical medicine) for possible filaricidal activities. The application of biochemicals to treat disease is among the oldest forms of healthcare known to humankind. Ancient Chinese characters and Egyptian papyrus hieroglyphs have documented the use of herbal medicine dating back to as early as 3000 B.C. [5]. In fact, herbal remedies had been in use by all cultures throughout the human history.

In recent years, it is not uncommon to find literature reported on the use of biochemical extracts against lymphatic filariasis [6–11]. Biological extracts such as those from *Azadirachta indica* [12], *Polyalthia suaveolens* [13], *Andrographis paniculata* [14], *Bauhinia racemosa* [15], and *Haliclona oculata* [16] were reported to have a bioactivity on either the filarial parasites or thier vectors. These extracts were believed to exert their bioefficacy through immunomodulatory elicitation of Th1/Th2 response, either by single (Th1, Th2) or mixed adjuvant activity. This paper presents a detailed review on the recent literature that reported the efficacy of direct filaricidal biomedicine or those employed as vector control agents. In addition, methods used for biochemical extraction, screening procedures, biochemical structure of the bioactive compounds and attempts made by researchers to evaluate the efficacy of the bio-chemical extract were also discussed.

## 2. Biomedicinal Agents Used against Filarial Parasites 

### 2.1. *Azadirachta indica* (Family: Meliaceae)

**Figure 2:**
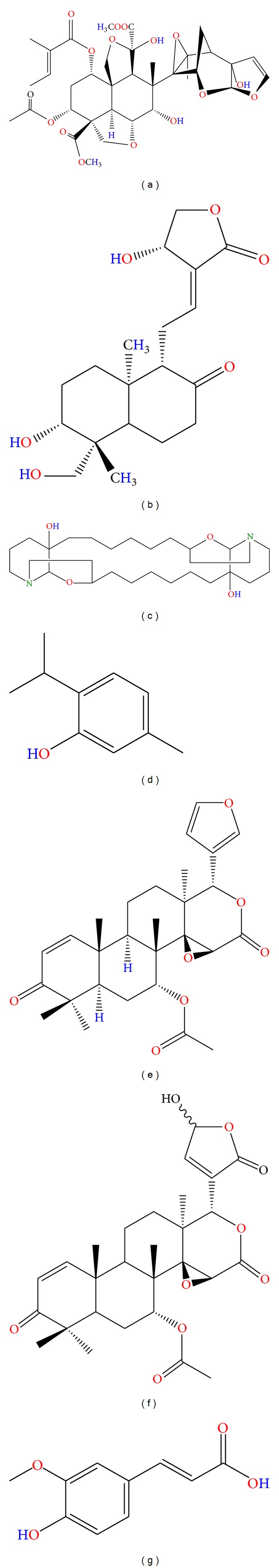
Chemical structure of (a) azadirachtin [19], (b) andrographolide [25], (c) araguspongin C [26], (d) 2-isopropyl-5-methyl phenol [27], (e) gedunin [28], (f) photogedunin [28], and (g) ferulic acid [29].

Popularly called “neem tree,” the plant is a large evergreen tree with its height reaching up to 50 ft. [17]. It is widely distributed within the hot tropical regions especially in India and West Africa [18]. The leaves and bark of this tree have a bitter taste of triterpenoid bio-chemical component described as azadirachtin (Figure 2(a)) [19]. Almost every part of this tree is reported to be used in complementary medicine for the cure of different ailments such as antimicrobial [20], anti-inflammatory [21], anticancer [22], antimalarial [23], antiulcerogenic [24], and antifilarial [17] activities. Al-Rofaai et al. [12] reported the effect of *A. indica* leaf extract against the helminth *Teladorsagia circumcincta *(Table 1). Employing organic solvents extraction and aqueous fractionation methods, they found that the first stage larvae (L_1_) were shown to be more sensitive having the lowest LC_50_ at 7.15 mg/mL of the extract as compared to 24.91 mg/mL on infective stage larvae (L_3_). Other workers employing distilled alcoholic and aqueous extracts of *A. indica* flowers showed that they have potential antifilarial activity against microfilariae of *Setaria cervi* [17]. The study also showed that the inhibition was concentration dependent, and both extracts were found to have almost similar lethal effect on the microfilariae of *S. cervi*, with LC_50_ being 15 and 18 ng/mL, respectively [17].

### 2.2. *Andrographis paniculata* Linn. (Family: Acanthaceae)

*Andrographis paniculata *(Kalmegh) is an annual herbaceous plant belonging to the family Acanthaceae, native to Southeast Asia especially China, India, and Sri Lanka. It has been traditionally used for centuries in Ayurvedic medicine. The herb has been revered for treating infectious diseases and highly regarded as having a preventative effect from many diseases, due to its powerful immune strengthening benefits [35]. Extensive research literature has revealed that *A. paniculata* has a broad range of pharmacological activities in different ailments such as being antianalgesic [36], antioxidant [37], antibiofilm [38], gastroprotective [39], wound healer [40], hepatoprotective [41], antifilarial [14], antimicrobial [42, 43], anticancer agent [44], antimalarial [45], and antitermitic [46]. It has been reported that the prophylactic effect of *Andrographis* was its ability to stop the catastrophic effect of the deadly flu virus of 1919 global epidemic from reaching India [47]. In fact, its bioactive diterpenoid andrographolide (Figure 2(b)) and its analogs were reported to block the MCF-7 breast cancer cells cycle at the G0-G1 phase [25].

Kumarappan et al. [48] studied the antifilarial activity of alcoholic extract of *A. paniculata*. Another study reported that aqueous extract of the leaves showed microfilaricidal activity on *Dipetalonema reconditum* within 40 min (Table 1), both *in vitro* and *in vivo *[30]. Administration of the extract (0.06 mL/Kg body weight) reduced the number of the microfilariae in infected dogs by more than 85% [30]. Earlier, Zaridah et al. [31] reported the filaricidal activity of *A. paniculata* aqueous leaf extract against *B. malayi*. The authors analyzed the antifilarial activity of the extract using relative movability (RM) value of the adult worm over a period of 24 hr. The use of 5 or 10 mg/mL of the extract resulted in 0% of RM value signifying total death of the parasite. Lowering the concentration of extract to 1 mg/mL, however, failed to produce similar effect (mean RM value was 35%). 

### 2.3. *Haliclona* sp. (Family: Chalinidae)

*Haliclona oculata *does not belong to plant kingdom; it is a marine demospone of family Chalinidae in the animal kingdom. It is known to possess a variety of bioactivities against many diseases such as cancer [49], neurodegeneration [50], type 2 diabetes [51], and fungal and microbial infections [52, 53]. The biological activity of these sponges is said to be due to the presence of novel sterols, metabolites including steroids, terpenoids, alkaloids, cyclic peptides, and unsaturated fatty acids [16]. Gupta et al. [16] reported the antifilarial activity of *H. oculata* against experimental lymphatic filaria *B. malayi*. Employing methanolic extract, chloroform fraction of the methanolic extract, and a fraction from the chromatographic eluent, at 100 mg/kg for five consecutive days by subcutaneous route, demonstrated significant macrofilaricidal efficacy of 51.3%, 64%, and 70.7%, respectively. In all the samples, about 45–50% macrofilaricidal activity with moderate embryostatic effect was observed. Further analyses on the chromatographic fraction revealed that it contained a mixture of four alkaloids, namely, mimosamycin, xestospongin C, xestospongin D, and araguspongin C together with few minor compounds [16]. Work done by Lakshmi et al. [26] on antifilarial activity of another species, *H. exigua* against lymphatic *B. malayi in vitro* and *in vivo *study, showed that 31.25 *μ*g/mL concentrations of the crude methanolic extract and butanol soluble fraction were able to kill the adult worm, whereas the chloroform extract was found to be effective at lower concentration (15.6 *μ*g/mL). According to the authors, such finding could be attributed to the single bioactive molecule “araguspongin C” (Figure 2(c)).

### 2.4. *Trachyspermum ammi* (Family: Apiaceae)

*Trachyspermum ammi* (Ajwain) is a native of Egypt and is cultivated in Iraq, Iran, Afghanistan, Pakistan, and India [54]. It has been reported to possess various pharmacological activities like being anti-fungal, antioxidant, antimicrobial, antinociceptive, cytotoxic, hypolipidemic, anti-hypertensive, anti-spasmodic, bronchodilating, anti-lithiasis agent, diuretic, abortifacient, antitussive, nematicidal, anti-helmintic, and antifilarial [54]. Studies on its bio-chemical composition and characterization revealed the presence of various bio-chemical constituents, mainly carbohydrates, glycosides, saponins, phenolic compounds, volatile oil (thymol, *γ*-terpinene, para-cymene, and *α*- and *β*-pinene), protein, fat, fiber, and mineral matter containing calcium, phosphorous, iron, and nicotinic acid [54]. Mathew et al. [27] studied the effect of methanolic extract of *T. ammi* fruits against adult bovine filarial *Setaria digitata* worms at a concentration of 0.01–0.5 mg/mL for a period of 24–48 hr (Table 1) and found that both the crude extract and the active fraction showed significant activity against the adult *S. digitata* by both worm motility and MTT (3-(4,5-dimethylthiazol-2-yl)-2,5-diphenyltetrazolium bromide) reduction assays. They also isolated a compound and characterized it as phenolic monoterpene described as 2-isopropyl-5-methyl phenol (Figure 2(d)). When tested *in vivo *for antifilarial activity against the human filarial worm *B. malayi* in *Mastomys coucha*, it presented a macrofilaricidal activity where female worm sterility was detected.

### 2.5. *Ricinus communis* (Family: Euphorbiaceae)

*R. communis* (castor oil plant) is commonly found in both the tropical and temperate climates of the world. The seed extract was reported to have efficacy in the treatment of warts, cold tumours, indurations of the mammary glands, corns, and moles, as well as being widely used as a human laxative-cathartic agent [32]. Ramanathan and Shanmugapriya [32] observed the filaricidal effect of organic solvent extract of *R. communis* seed against filarial parasite *B. malayi* using different dosages (10, 50, and 100 *μ*g/mL of the extract) for the period of 24 hours. Their findings indicated dose-dependent filaricidal activity (40–90%). Nisha et al. [33] reported that treatment with ethanol fraction (1 mg/mL) of *R. communis* seed extract caused a complete suppression of *S. digitata* microfilarial growth within 1 hr and 40 min (Table 1). Based on worm motility and MTT reduction assay, the authors found the seed extract to cause about 72.39% growth inhibition within 4 hr of exposure [33].

### 2.6. *Morinda citrifolia* L. (Family: Rubiaceae)

*M. citrifolia* (noni) is plant that could be found in a wide variety of habitats including volcanic terrains, lava-strewn coasts, and clearings or limestone outcrops. It can grow up to 9 m (30 ft) tall due to its tolerance to adverse agricultural conditions such as saline soils, drought conditions, and secondary soils. It has large, simple, dark green, shiny, and deeply veined leaves. The medicinal applications of this plant span over all kinds of ailments involving both modern and complementary medicines. Satapathy [34] reported the filaricidal activity of noni against *W. bancrofti* in an *in vitro* study. The study showed that adding noni fruit extract to culture media containing *W. bancrofti* microfilariae at a concentration of 2000 microfilaria per mL (Table 1) killed the parasite within 20 hours as compared to the control group without adding the extract, which survived for up to 60 hours [34].

### 2.7. *Xylocarpus granatum* (Family: Meliaceae)

*Xylocarpus granatum* (nyireh bunga) is a mangrove swamp species with reported medicinal importance [31]. Misra et al. [28] found an *in vivo *filaricidal activity of *X. granatum* extract against *B. malay*i (both adult worm and microfilaria). On testing the crude aqueous ethanolic extract *in vitro*, they observed IC_50_ of 15.46 and 13.17 *μ*g/mL in both adult worm and microfilariae, respectively. On the other hand, study on the ethyl acetate-soluble fraction revealed the antifilarial activity to be moderate (IC_50_ of 8.5 and 6.9 *μ*g/mL) in both adult and microfilariae, respectively. They further found that on testing the extract's efficacy *in vivo* by administering it to Mastomys orally at 50 mg/Kg, it showed adulticidal (52.8%) and embryostatic (62.7%) effects against *B. malayi*. Isolation of the bioactive biochemical components of this plant revealed eight pure molecules with two of these compounds, namely, gedunin (Figure 2(e)) and photogedunin (Figure 2(f)), at 100 mg/kg by subcutaneous route revealing excellent adulticidal efficacy resulting in the death of about 80% *B. malayi* [28]. Previously, this species has been reported to have filaricidal activity against *B. pahangi* [55]. Filaricidal activity of *X. granatum* was further evaluated somewhere else.

### 2.8. *Hibiscus* sp. (Family: Malvaceae)

*H. sabdariffa* (roselle) is a native of tropics, used for the production of fiber and infusions that are normally used as beverages. It is a woody shrub of annual to perennial seasoning. It is reported to have variant medicinal efficacy, especially on hypertensive patients [56]. The plant is said to be rich in anthocyanins, as well as dihydroxybenzoic acid. Daphniphylline forms the major pigment, while the dried calyces contain the flavonoids gossypetin, hibiscetine, and sabdaretine. In addition, small amounts of delphinidin-3-monoglucoside, cyanidin-3-monoglucoside, and delphinidin were also present [56]. Moreover, the seeds were reported to be a good source of lipid-soluble antioxidants, particularly gamma-tocopherol [57]. Recently, Saxena et al. [8] reported for the first time the filaricidal activity of ethanolic extract of *H. sabdariffa* leafs against *B. malayi* (Table 1). The efficacy of the plant extract filaricidal activity was assessed using both the *in vivo *and *in vitro* motility and MTT reduction assays on the microfilariae (mf) and adult worms. The authors found that administering the leaf extract at 500 mg/mL for 5 days incurred about 30% macrofilaricidal efficacy and 42% sterilization of female worms in *Meriones unguiculatus*. On the other hand, feeding *M. coucha* with the extract (1 g/kg for 5 days) exerted 57.0% macrofilaricidal activity with 64% sterilizing effect on female worms [8]. In similar studies, the crude methanolic extract of *H. mutabilis* (confederate rose) and the isolated bioactive molecule “ferulic acid” were tested against bovine *S. cervi *[29]. The authors reported that both the extract and the bioactive molecule “ferulic acid” (Figure 2(g)) showed significant microfilaricidal as well as macrofilaricidal activities against *S. cervi*. Using 500 *μ*g/mL of the aqueous fraction of ethyl acetate extract, the authors reported about 50% microfilarial motility inhibition within 24 hours and more than 80% within 48 hours, while adult worms motility inhibition was observed to be about 40% and 70% within 24 and 48 hours, respectively. Test on the bioactive compound ferulic acid was shown to be more effective. At a concentration of 400 *μ*g/mL, it caused 100% and 90% motility inhibition in both micro- and macrofilariae after 48 hours, respectively [29]. 

### 2.9. *Cardiospermum halicacabum* (Family: Sapindaceae)

*Cardiospermum halicacabum* (love in a puff) is a climbing plant widely distributed in tropical and subtropical regions of Africa and Asia [58]. This plant has been reported to have bioactivity, such as being homoeopathic [58], having anti-diarrheal efficacy [59], and being antimicrobial [60, 61]. Khunkitti et al. [62] previously reported the *in vitro* filaricidal activity of ethanolic and aqueous extracts of this plant against *B. pahangi*. The researchers found activity on the adult worms and the amount of microfilariae released by female worms was concentration and time dependent. For example, using 500 *μ*g/mL (Table 1), the authors observed that the aqueous extract significantly reduces motility of adult females after 24 h of exposure, the release of microfilariae from female worms on day 2, and the motility of the adult males after 3 days. However, the aqueous extract at this concentration (500 *μ*g/mL) did not affect the motility of microfilariae, with the exception of those in higher concentration extracts. In contrast, 500 *μ*g/mL of the ethanol extract was found to rapidly reduce the motility of microfilariae on day 2. Furthermore, higher concentrations of ethanol extracts (2 mg/mL) inhibit both the motility of adult worms and the release of microfilariae from females [62].

### 2.10. *Excoecaria agallocha* L. (Family: Euphorbiaceae)

*Excoecaria agallocha *is a small tree species that inhabits the mangrove swamps of Southeast Asia and that can grow up to 15 m height. The tree has a well-developed chemical defense mechanism composing of diterpenoids, triterpenoids, and flavonoids [63]. The extract of this tree is reported to possess depressant action on the central nervous system [64], antimicrobial efficacy [65], and anti-viral and anti-carcinogenic activities [64]. Patra et al. [66] reported the antifilarial activities of methanolic and aqueous extracts of *E. agallocha* leaf against *S. digitata* were dose dependent at concentrations of 10, 50, and 100 *μ*g/mL for 24 hrs at 37°C in 5% CO_2_ incubation. The study showed reduction in percentage of motility by about 20, 60, and 83%, respectively. Testing the radical scavenging activity of the extract, the authors found the aqueous extract to be effective in 2,2-diphenyl-1-picrylhydrazyl (DPPH) radical scavenging activity, reducing power, and hydrogen peroxide scavenging activity, which increased with the increase in concentration of the extract. Based on their observations, the authors concluded that *Excoecaria agallocha* can be a potential source of bioactive chemicals that can be used not only for meeting the oxidative stress generated during chronic manifestation of lymphatic filariasis in human beings but also for blocking embryogenesis in filarial parasites, which in turn can potentially affect their transmission and survival in host communities [66].

### 2.11. *Alnus nepalensis* (Family: Betulaceae)

**Figure 3:**
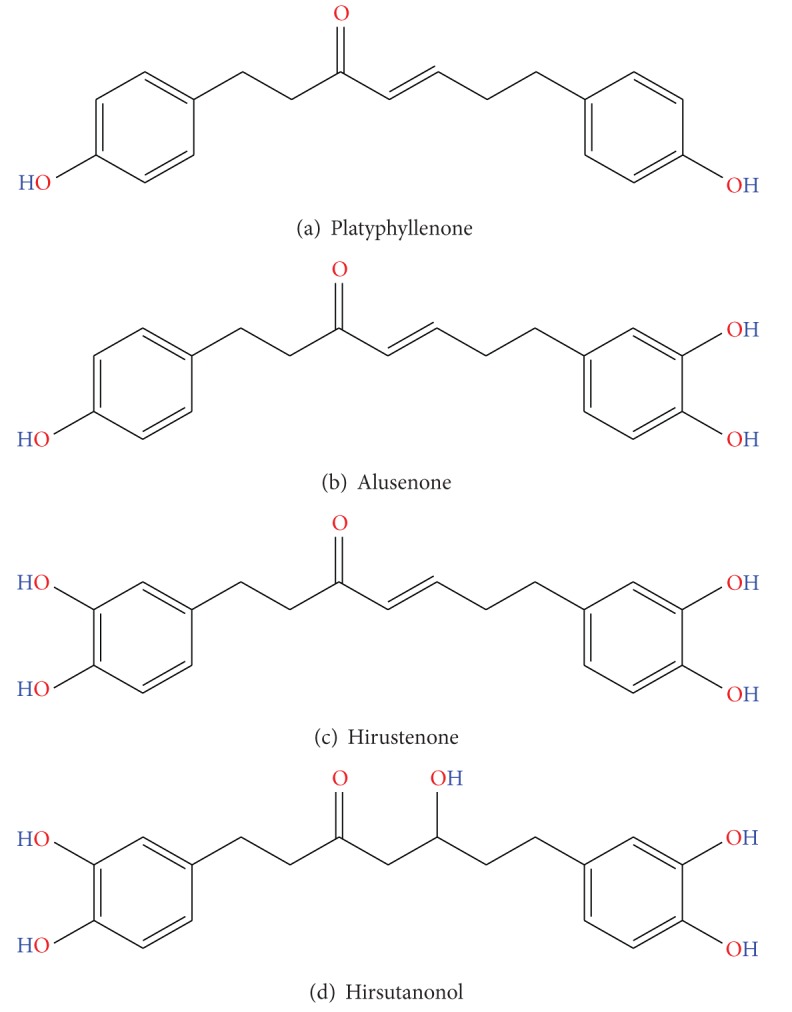
Chemical structure of bioactive biochemicals extracted from *A. nepalensis *[11].

**Figure 4:**
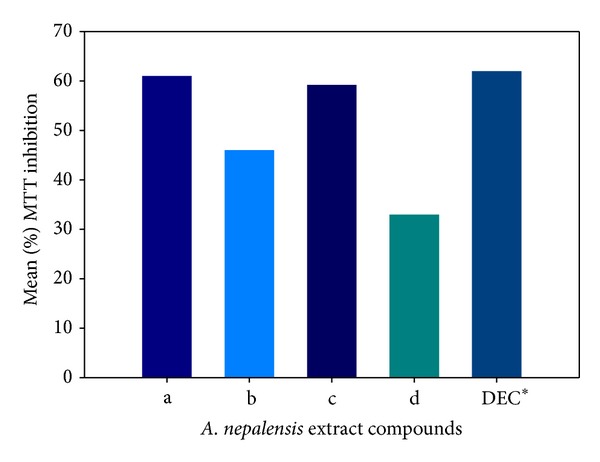
Effect of *A. nepalensis *extract on adult female worm motility inhibition, DEC* (diethylcarbamazine used as control).

*Alnus nepalensis* (Nepalese Alder) is widely found in the subtropical highland of Himalaya. It is a fast growing deciduous tree that reaches up to 30 m in height, and it can grow on different kinds of soils especially in wet areas. The leaves are naturally shallow with dimension of 7–16 cm long and 5–10 cm broadness. Occasionally, the tree is used for making boxes, in light construction, and as firewood by the local people. Furthermore, it is sometimes planted as erosion control on hillsides and for land recovery in shifting cultivation. Recently, the *in vitro* and *in vivo *filaricidal activities of four bioactive compounds isolated from *A. nepalensis* leaves against human lymphatic filariasis (*B. malayi*) have been investigated for the first time [11]. The researchers designated the isolated diarylheptanoid biochemicals as compounds (a–d), that is, (a) platyphyllenone, (b) alusenone, (c) hirustenone, and (d) hirsutanonol, as shown in Figure 3. The authors reported that compounds (a) and (c) showed better efficacy as indicated by about 60% mean inhibition in motility of adult worms (Figure 4). Comparing the mean percentage MTT inhibition with the control synthetic drug diethylcarbamazine (DEC) in Figure 4, the authors concluded that *A. nepalensis* extract especially compound (c) showed a promising antifilarial activity against both the macro- and microfilarial worms of *B. malayi*.

### 2.12. *Bauhinia racemosa* Lam. (Family: Fabaceae)

**Figure 5:**
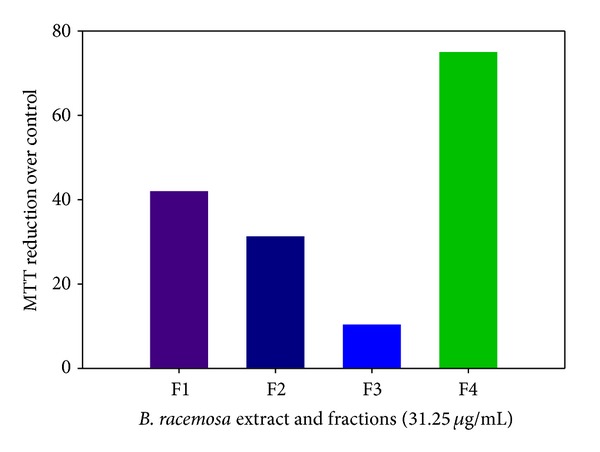
*In vitro* motility inhibition by MTT reduction assay as a function of *B. racemosa *extract and fractions exposure (F1: crude ethanolic extract; F2: n-hexane fraction; F3: chloroform fraction; F4: n-butanol fraction).

**Figure 6:**
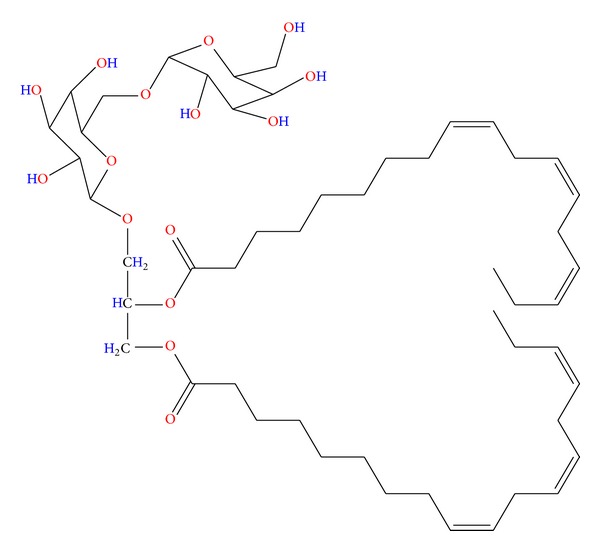
Chemical structure of the bioactive biochemical extracted from *B. racemosa *[15].

*Bauhinia racemosa* (mountain ebony) is a small deciduous tree native to the tropics especially Southeast Asia. Almost each and every part of this tree has some medicinal values [67]. The stem bark of the tree has analgesic activity [68], antidiabetic activity [69], and anti-helmintic activity [70] and is used in the treatment of headache, fever, skin diseases, blood diseases, dysentery, and diarrhea [67]. An extract of the leaves has been reported to show anti-histaminic effect [71], antimicrobial efficacy [72], and hypolipidemic activity [73], while a decoction of the dried fruits has antiulcer activity [74]. The tree is demonstrated to have antioxidant and hepatoprotective effects [75]. Sashidhara et al. [15] recently reported the filaricidal activity of the galactolipids compounds isolated from ethanolic extract of *B. racemosa* leaves. Results were based on the fractions tested (Figure 5); the n-butanol fraction of the extract revealed promising adulticidal (IC_50_ 5.46 mg/mL) and microfilaricidal (IC_50_ 4.89 mg/mL) activity, with minimum inhibitory concentration (MIC) of 15.6 mg/mL [15]. Among the characterized isolated galactolipid is *(2S)-*1,2-di*-O-*linolenoyl*-3-O-*a-galactopyranosyl*-(1/6)-O-*b-galactopyranosyl glycerol (Figure 6), which they found to have filaricidal efficacy that rivals the standard drug ivermectin (IC_50_ 1.61 mg/mL; MIC 7.8 mg/mL in adult and IC_50_ 3.62 mg/mL; MIC 125 mg/mL in microfilariae) in terms of dose and efficacy [15].

### 2.13. *Cocos nucifera* (Arecaceae)

The coconut palm is widely distributed within the tropical and subtropical regions, growing up to 30 meters tall, with pinnate leaves 4–6 meters long. The tree has versatile importance spanning from domestic, commercial to medicinal applications. The biochemical analysis of the endosperm revealed the presence of terpenoids, alkaloids, resins, glycosides, steroids, and flavonoids [76, 77]. Medically, coconut is reported to have efficacy against prostatic hyperplasia [78], anti-helmintic activity [79], and antimicrobial and antiviral activities [80], vasorelaxant, antimalarial [81], and anti-hypertensive; activities, and inhibitory effect on oral microflora [82]. Furthermore, its bioactive compounds were found to have extended efficacy on agropest control [83]. Al-Adhroey et al. [81] studied the antimalarial efficacy of *C. nucifera* endocarp methanolic extract against *Plasmodium berghei *(NK65) infections in mice. The antimalarial activity was evaluated using different doses (50, 100, 200, and 400 mg/kg) of the extract in reference to chloroquine (20 mg/kg) and pyrimethamine (1.2 mg/kg) drugs. Although, at 200 and 400 mg/kg doses, the extract revealed significant reduction of parasitaemia, it failed to increase the survival time of the infected mice.

## 3. Medicinal Plants Used as Agents for Vector Control 

Among the methods used to manage filariasis is control its vectors, since filariasis is transmitted by mosquito vectors of the genera *Aedes*, *Anopheles*, *Culex*, and* Mansonia*. In this section, a review on the recent use of bio-chemical extracts in the control of these filarial vectors is presented.

*Cocos nucifera (Arecaceae)*. Recently, Roopan et al. [84] employed the use of novel biosynthesis that reduced silver nitrate to biogenic silver nanoparticles in the presence *C. nucifera* extracts against *A. stephensi* and *C. quinquefasciatus*. The researchers reported about 100% and 92% 4th instar larval motility inhibition at 4 mg/mL dosage in both *A stephensi* and *C. quinquefasciatus* after 72 hours, respectively. 

Subarani et al. [85] observed the larvicidal activity of *Vinca rosea *(Apocynaceae) aqueous leaf extract biosynthesized silver nanoparticles also against *A. stephensi *Liston and *C. quinquefasciatus*. On exposure for 72 hours, the researchers found that the larvicidal activity showed maximum efficacy in synthesized silver nanoparticles against the fourth instar larvae of *A. stephensi *(LC_50_ = 16.84 mg/mL) and against *C. quinquefasciatus* (LC_50_ = 43.80 mg/mL).

**Figure 7:**
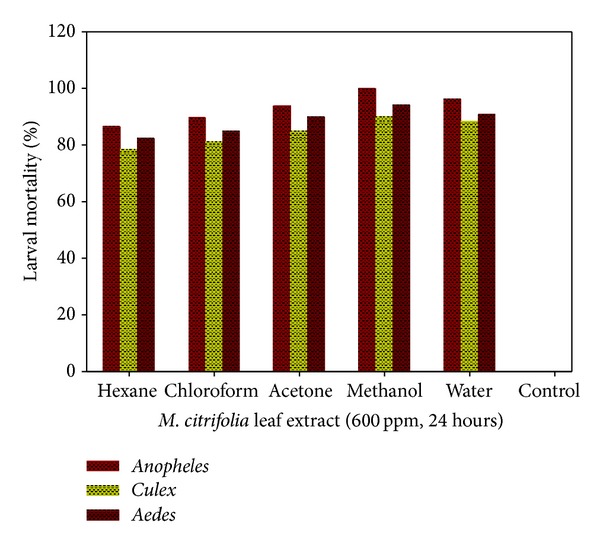
*M. citrifolia *solvent extract as a function of percent larval mortality.

Kovendan et al. [86] recently reported the larvicidal effect of *M. citrifolia* leaf extract against three medically important mosquito vectors of *Anopheles*, *Aedes,* and *Culex* genera. After 24 hours of exposure, the authors found that the larvicidal activity was concentration and extraction-solvent dependent (Figure 7).

Govindarajan et al. [87] reported the larvicidal and ovicidal activity of *Cassia fistula* Linn. (Fabaceae) methanolic leaf extract against *C. quinquefasciatus* and *A. stephensi*. The researchers found the activity of the extract (10–50 mg/L) was concentration dependent. At 40 mg/L the percent mortality was 89.33 and 100 in both *Culex* and *Anopheles*, respectively, with LC_50_ values recorded as 17.97 and 20.57 mg/L, respectively, signifying that the extract is more potent to *Anopheles* than to the *Culex* larvae. Their investigation on the ovicidal activity of the extract, based on percentage of hatchability, showed that it was inversely proportional to the concentration (25–200 mg/L) used. The authors also investigated the bioefficacy of *C. fistula* leaf extract on *A. aegypti*. They reported that the activity was concentration and extraction-solvent dependent. In methanol extract, exposure to 25 mg/L for 24 hours resulted in 98% larval mortality. The percentage of mortality was reduced up to about 31% when the concentration was brought down to 5 mg/L. Exposure to 20 mg/L of methanol extract for 24 hours resulted in about 85% larval mortality. By using the same concentration, only 55% and 41% larval mortality were observed when employing benzene and acetone extracts, respectively.

 In contrast to the earlier study, Rajkumar and Jebanesan [88] reported LC_50_ value of 52.2 mg/L in *A. stephensi* using ethanolic leaf extract of Chinese Senna (*Cassia obtusifolia* Linn.; Fabaceae). As for ovicidal activity, the researchers used an extract concentration of 100–400 mg/L, and they found the oviposition deterrent activity of the leaf extract to be concentration dependent. At extract concentration of 400 mg/L, oviposition effective repellency of 92.5% was indicated while at 300, 200, and 100 mg/L the effective repellency of 87.2%, 83.0%, and 75.5% was observed, respectively.

Recently, the bioefficacy of *Carica papaya *(Caricaceae) leaf extracts against *A. aegypti* larvicidal and pupicidal properties was reported [89]. The authors found that the leaf extract showed both larvicidal and pupicidal types of efficacy after 24 h of exposure. In all the extracts tested, the methanolic extract has the highest larval and pupal mortality against the larvae and pupae with values of LC_50_ of 51.76, 61.87, 74.07, 82.18, and 440.65 ppm for 1st, 2nd, 3rd, and 4th instar larvae and pupae, respectively. 

**Figure 8:**
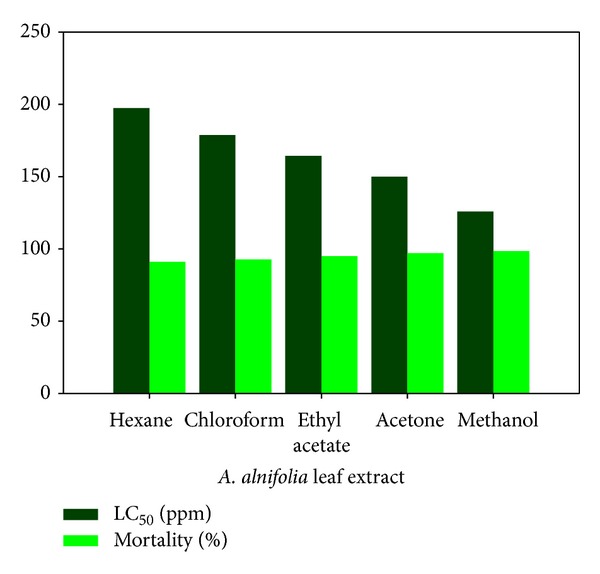
Influence of extraction solvent on larval percent mortality and LC_50_ during exposure to *A. alnifolia *extract.

The author also evaluated the larvicidal activity of *Acalypha alnifolia* Klein ex Willd. (Euphorbiaceae) leaf extract against *A. stephensi*, *A. aegypti,* and *C. quinquefasciatus* [90]. Exposing the larvae to extract of different solvents for 24 hours, methanol extract was shown to be the most bioactive (98.4% larval mortality) while hexane extract was found to be the least bioactive as shown in Figure 8. 

A combined bioefficacy of the fruit extracts of *Solanum xanthocarpum* and copepods of *Mesocyclops thermocyclopoides* was assessed for the control of *A. aegypti*, respectively [91]. The *S. xanthocarpum* fruit extract revealed significant larval mortality to *A. aegypti* 1st–4th instar larvae exposed to dosage of 100–300 ppm (the highest LC_50_ = 253.18 ppm). The authors reported an increase in the percentage of copepod predatory efficiency in the extract treated sample (8.7%) as compared to 6.5 % in samples without the extract. This increase in predation efficiency was opined by the authors to possibly be due to the detrimental effects of the extract active principle compound (solanocarpine and solanocarpidine) on the mosquito larvae [91]. 

Lalrotluanga et al. [92] recently reported the larvicidal, adulticidal, and repellent activity of *Hiptage benghalensis* L. Kruz (Malpighiaceae) acetone root bark extracts against mosquitos of *Anopheles*, *Culex,* and *Aedes* genera. The extract was found to be effective as larvicide with low LC_50_ (11.15–16.78 ppm) and lethal time LT_50_ (1.25–4.84 h at 200 and 400 ppm). The lethal time was found to be concentration dependent. 

Kovendan et al. [93] reported their work on the larvicidal efficacy of *Sphaeranthus indicus*, *Cleistanthus collinus,* and *Murraya koenigii* organic-solvent leaf extracts against the third instar larvae of *C. quinquefasciatus*. Using a dosage of 750 ppm, significant mortality of larvae was observed. *S. indicus* extracts showed mortality of 78.62% in chloroform, 100% in ethyl acetate, and 60.16% in hexane extracts within 72 hours. *C. collinus* extract, on the other hand, showed 100% mortality in chloroform and 78.09% in hexane. Exposure to *M. koenigii* extract for 72 hours showed mortality of 91.24% (ethyl acetate), 89.03% (chloroform), and 86.35% (hexane), respectively [93].

*Indigofera suffruticosa* Mill. (Fabaceae) is a plant found in tropical and subtropical areas and well adapted to growth in semiarid regions and low fertile soil. The plant has been known for its medicinal efficacy against bacterial and fungal infections [94]. Vieira et al. [94] reported their study on the oviposition and embryotoxicity of *I. suffruticosa* leaves extract against *Aedes aegypti.* The authors found the repellent activity to be concentration dependent. Exposing the larvae to concentration of 250 *μ*g/mL for 72 hours showed 93.3% growth inhibition in L2 instar [94].

## 4. Conclusion

Ancient biomedicine described the use of plants in traditional system of medicine for the treatment of several human ailments, including filarial infections. This kind of complementary medicine provides an avenue for therapeutic treatment in a more benign approach, with plant materials that are mostly available and easily assessable. The present report is a survey of literature indicating the screenings of crude plant extracts, essential oils, and isolated active principles for *in vitro* and *in vivo *filaricidal activities to substantiate those folklore claims. It is worth mentioning that despite the fact that infection with *W. bancrofti* accounts for major incidence cases (91%) of total lymphatic filariasis infections while *B. malayi* and *B. timori* are responsible for only 9% in South and Southeast Asia, literature on biomedicinal efficacy against bancroftian filariasis is highly scarce; specifically the research on that area seems to be neglected. Hence, we opined that, in future studies, more research on filaricidal biochemicals with efficacy on *W. bancrofti* is needed. 

Furthermore, proper control of filarial vector can be achieved via careful design of extraction and administration processes such as use of efficient bio-chemical solvent extraction, preferably hydrophilic solvent, and logically controlled dosage.

## Figures and Tables

**Table 1:** Bioproduct with reported filaricidal activity.

Name	Family	Part used	Extraction method	Filarial pathogen	Dosage	Reference
*Azadirachta indica *	Meliaceae	Leaf	Organic solvent and water fractionation	*Teladorsagia circumcincta *	3.1–50 mg/mL	[12]
		Flower	Distilled ethanol Distilled water	*Setaria cervi *	5–25 ng/mL	[17]
*Andrographis paniculata *	Acanthaceae	Leaf	Water decoction	*Dipetalonema reconditum*	0.06 mL/Kg	[30]
		Leaf	Aqueous extract	*B. malayi *	0.5–10 mg/mL	[31]
*Heliclona oculata *	Chalinidae	Sponges	Methanol extract solvents fractionation	*B. malayi *	100 mg/Kg	[16]
*Haliclona exigua *	Chalinidae	Sponges	methanolic extract and butanol fraction	*B. malayi *	15.6–31.2 *µ*m/mL	[26]
*Trachyspermum ammi *L.	Apiaceae	Fruits	Methanolic extract	*Setaria digitata* *B. malayi *	0.01–0.5 mg/mL	[27]
*Ricinus communis *	Euphorbiaceae	Seed	Methanolic extract ethanol fractionation	*B. malayi* *S. digitata *	10–100 *µ*g/mL 1 mg/mL	[32] [33]
*Morinda citrifolia *	Rubiaceae	Fruits	Aqueous extract	*W. bancrofti *	0.02–0.04 noni : media	[34]
*Xylocarpus granatum *	Meliaceae	Leaf	Ethanolic extract Aqueous extract	*B. malayi* *B. pahangi *	100 mg/kg 0.5–10 mg/mL	[28] [31]
*Hibiscus sabdariffa *	*Malvaceae *	Leaf	Ethanolic extract	*B. malayi *	500 mg–1 g/mL	[8]
